# AMPK Inhibition Blocks ROS-NFκB Signaling and Attenuates Endotoxemia-Induced Liver Injury

**DOI:** 10.1371/journal.pone.0086881

**Published:** 2014-01-24

**Authors:** Yuan Guo, Yi Zhang, Kai Hong, Fengyan Luo, Qiuping Gu, Nonghua Lu, Aiping Bai

**Affiliations:** 1 Department of Gastroenterology, the First Affiliated Hospital of Nanchang University, Nanchang, China; 2 Department of Pharmacy, the First Affiliated Hospital of Nanchang University, Nanchang, China; Boston University School of Medicine, United States of America

## Abstract

**Background:**

AMP-activated protein kinase (AMPK) is an important enzyme in regulation of cellular energy homeostasis. We have previously shown that AMPK activation by 5-aminoimidazole-4-carboxamide (AICAR) results in suppression of immune responses, indicating the pivotal role of AMPK in immune regulation. However, the cellular mechanism underpinning AMPK inhibition on immune response remains largely to be elucidated. The study aimed to investigate the effects of AMPK inhibition on reactive oxygen species (ROS)-nuclear factor κB (NFκB) signaling and endotoxemia-induced liver injury.

**Methodology/Principal Findings:**

RAW 264.7 cells were pretreated with AMPK activator or inhibitor, followed by LPS challenge. In addition, LPS was injected intraperitoneally into mice to induce systemic inflammation. The parameters of liver injury and immune responses were determined, and survival of mice was monitored respectively. LPS challenge in RAW 264.7 cells resulted in AMPK activation which was then inhibited by compound C treatment. Both AMPK activation by AICAR or inhibition by compound C diminished LPS-induced ROS generation, inhibited phosphorylation of IKK, IκB, and NFκB p65, and consequently, decreased TNF production of RAW 264.7 cells. AICAR or compound C treatment decreased ALT, AST, and TNF levels in serum, reduced CD68 expression and MPO activity in liver tissue of mice with endotoxemia. Moreover, AICAR or compound C treatment improved survival of endotoxemic mice.

**Conclusions:**

AICAR or compound C treatment attenuates LPS-induced ROS-NFκB signaling, immune responses and liver injury. Strategies to activate or inhibit AMPK signaling may provide alternatives to the current clinical approaches to inhibit immune responses of endotoxemia.

## Introduction

Endotoxemia is one of the critical clinical diseases with high mortality and poor prognosis. As the most potent microbial mediators implicated in endotoxemia, bacterial components such as lipopolysaccharide (LPS), also termed endotoxin, can initiate excessive activation of immune cells, and induce large amounts of proinflammatory cytokine and chemokine productions, which results in shock and multiple organ injury [1]. Currently, immune cells such as macrophages and neutrophils, are recognized as primary pathogenic cells playing an important role in tissue damage, organ injury, and death in humans and other animals during the process of endotoximia [2], [3]. Inhibition of immune cell function has emerged as a target for endotoximia treatment.

The intracellular signals in control of immune cell function have been intensively studied. Among those signals, nuclear factor (NF) κB is a transcription factor responsible for inflammation and immunity of mammals and human beings [4]. Once activated by LPS and other extracellular stimuli, the dimer of NFκB composed of the P65 and P50 subunits translocates to nucleus and regulates a variety of target genes in immune cells, including multiple inflammatory cytokines and chemokines, resulting in amplification of inflammatory responses and tissue damage [4], [5]. Recently, we and others have shown that dampening NFκB signal can attenuate immune responses, and ameliorate immune diseases in animals [6]–[8], supporting the beneficial effects of inhibition of NFκB signaling.

AMP-activated protein kinase (AMPK) is an important enzyme which plays an important role in the regulation of cellular energy homeostasis [9]. Recently, AMPK has been reported as an anti-inflammatory protein, and its natural ligand, 5-Aminoimidazole-4-carboxamide (AICAR), exhibits inhibitory effects on immune responses in intestine and lung in several animal models of inflammation [10], [11]. The current studies indicate the pivotal role of AMPK in immune regulation. It remains to be elucidated, however, if inhibition of AMPK can regulate NFκB signaling and modulate immune responses. Here, we report modulation of NFκB signaling in immune cells by AMPK inhibition. Both, activation and inhibition of AMPK, diminish reactive oxygen species (ROS)-NFκB signaling cascades inclusive of *IκB kinase* (*IKK*), inhibitor of κB (IκB), and NFκB, and protect mice against LPS induced endotoximia. Management especially inhibition of AMPK signaling may provide new approaches and strategies for the treatments of immune diseases including endotoxemia and other critical care conditions.

## Methods

### Cell Culture and Treatment

Murine macrophage cell line RAW 264.7 was cultured in HEPES-buffered Dulbecco’s modified Eagle’s medium (DMEM) containing 10% fetal bovine serum, penicillin G (100 U/mL), and streptomycin (100 µg/mL) at 37°C.

For in vitro study, about 1×10^6^/mL RAW264.7 cells were seeded in 48-well plates. The cells were treated with AICAR (Toronto Research Chemicals Inc) or/and compound C (Sigma-Aldrich) for different time points. Alternatively, the cells were pretreated with AICAR or/and compound C from 15 min to 1 hour, prior to 100 ng/mL or 1 µg/mL lipopolysaccharide (LPS, from Escherichia coli 0111:B4; Sigma-Aldrich) challenge.

### Reactive Oxygen Species (ROS) Detection

The cells were pretreated with 2 µM Carboxy-2′,7′-dichlorodihydrofluorescein diacetate (H_2_DCFDA) (Invitrogen) in the presence or absence of 10 µM diphenyleneiodonium chloride (DPI) (Sigma-Aldrich), AICAR, or compound C for 15 min, followed by LPS (1 µg/mL**)** stimulation. After 15 min, ROS generation was determined by flow cytometric analysis. The H_2_DCFDA-untreated cells were defined as negative control.

### Enzyme-linked Immunosorbent Assays (ELISA)

Tumor necrosis factor (TNF) levels in culture supernatants or serum of mice were determined by ELISA, following manufacturer’s instructions (R&D Systems, Inc). Briefly, polyclonal rat anti-mouse cytokine antibodies were used as capturing antibodies and biotinylated polyclonal rat anti-mouse cytokine antibodies for detection, and the standard curve of TNF was set up meanwhile. Color changes were determined at 450 nm.

### Western Blot

Proteins were extracted from the cultured cells using a lysis buffer (0.1 M PBS pH7.4 containing 1% deoxycholic acid sodium, 0.2% SDS, and protease inhibitors). After measurement of protein concentration, the proteins of the samples were loaded and separated by SDS-PAGE and then electrophoretically transferred to polyvinylidene difluoride membranes. The membranes were incubated with primary rabbit antibodies, which included anti-phospho-IKKα/β (Ser176/180), anti-phospho-IκBα (Ser32), anti-phospho-NFκB P65 (Ser536), anti-phospho-acetyl-CoA carboxylase (ACC) (Ser79), anti-phospho-AMPKα1 (Thr172), or anti-AMPKα1 (from Cell Signaling Technology), or mouse anti-β-actin antibody (from Santa Cruz Biotechnology). After incubating with secondary antibodies, the immunoreactive bands were visualized using the SuperSignal West Femto Maximum Sensitivity Substrate reagents (Thermo Scientific). Rabbit anti-β-actin was used as an inner control. Relative protein levels were evaluated by Image J software (NIH).

### Immunohistochemistry

Liver tissues of mice were taken and fixed immediately in 10% buffered formalin, embedded in paraffin, and cut into 4 µm sections. After blockade of inner peroxidase, sections were incubated sequentially with the first antibody solution including rabbit anti-CD68 antibody (Abcam). After three washes in PBS (pH 7·4), the sections were then incubated in secondary goat anti-rabbit immunoglobulin (Ig)G conjugated with peroxidase labeled polymer, prior to colorization using diaminobenzidine reaction and counterstained with haematoxylin. Negative controls were established using rabbit IgG instead of the first antibodies.

### Measurement of Myeloperoxidase **(**MPO) Activity

All experiments were performed within 1 week of tissue collection. MPO activity was measured according to the method previously described with minor modification [12], [13]. In short, tissues were homogenized in hexadecyltrimethylammonium bromide in 50 mM potassium phosphate buffer. Aliquots were then added to O-dianisidine hydrochloride solution. Absorbance was read at 450 nm using a microplate reader. MPO was expressed in units/gram tissue, where 1 unit corresponds to the activity required to degrade 1 mmol hydrogen peroxide in 1 min at 24°C.

### Animal Studies

Male BALB/c mice at 6–7 weeks of age weighing 20–22 g were fed with food and water *ad libitum*, and housed in a standard animal facility with 12 h light/dark cycle and 50%–70% humidity) for 3 days before the study. All animal experiments were performed to minimize animal suffering according to the Guide for the Care and Use of Laboratory Animals which was issued by the National Institutes of Health in 1996. All animal procedures were approved by the Institutional Animal Care and Use Committee of the First Hospital of Nanchang University.

BALB/c mice were randomly divided into five experimental groups: Control (intraperitoneally (i.p.) injection of PBS), LPS (i.p. injection of 2 mg/kg body weight), LPS+AICAR (i.p. injection, 500 mg/kg body weight 60 min before LPS injection), LPS+Compound C (CC) (i.p. injection, 25 mg/kg body weight 60 min before LPS challenge), and LPS+AICAR+CC (i.p. injection of 500 mg of AICAR and 25 mg of compound C per kilogram of body weight 60 min before i.p. injection of LPS). Six or twelve hours after injection of LPS, the mice were anesthetized with pentobarbital and euthanized thereafter by cervical dislocation, and blood and tissues were collected for analysis.

For survival experiment, the grouped mice as mentioned above were injected i.p. with LPS (20 mg/kg body weight). To investigate the effect of AICAR or compound C, the mice received injection (i.p.) of 500 mg of AICAR or/and 25 mg of compound C per kilogram of body weight 60 min before administration of LPS. Survival of animals was monitored every 2 hours for up to 24 hours. Severity of sepsis was monitored according to general appearance, breathing frequency, and provoked behavior. The mice were euthanized by cervical dislocation under deep anaesthesia, if the mice exhibited a disease point of no return. After 24 hours, the number of the survival mice of each group was recorded: 8 mice survived in total 8 Control mice (8/8), 0/20, 8/19, 12/19, and 3/19 surviving mice in LPS, LPS+AICAR, LPS+CC, LPS+AICAR+CC respectively. All surviving mice were anesthetized and euthanized with the same protocol described above.

### Statistical Analysis

The data were calculated as means ± SEM, and differences between experimental groups were assessed by One-way ANOVA, the Tukey-Kramer multiple comparisons test (for multiple groups), or Student’s t-test (for comparisons between 2 groups). For analysis of survival study, fisher’s exact test was used. *p*<0.05 was considered to be statistically significant.

## Results

### Compound C Blocks AICAR-induced AMPK Activation

**Figure 1:**
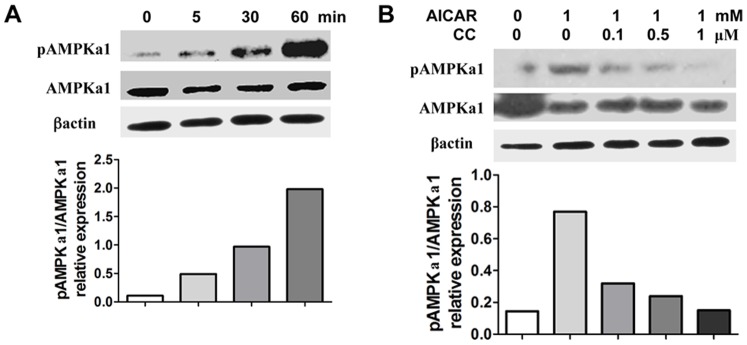
Compound C blocks AICAR induced AMPK activation. **A**. RAW 264.7 cells were treated with AICAR (1 mM) for different time points, and expression of phosphor-AMPKα1 (pAMPKα1) and AMPKα1 was determined by western blot. **B**. RAW 264.7 cells were treated with AICAR (1 mM) in combination with different doses of compound C (CC, 0, 1, 5, and 10 µM) for 30 min, and expression of pAMPKα1 and AMPKα1 was determined by western blot. Data are represented three to four independent experiments.

It has been recently recognized that AMPK signal controls inflammatory responses of immune disease models [14]. To study AMPK activation and phosphorylation induced by AICAR, one of its activators, we stimulated murine macrophage cells with AICAR, and observed AMPK activation induced by AICAR in a time-dependent manner (Fig. 1A), as well as a dose-dependent response [7].

Meanwhile, we treated RAW 264.7 cells simultaneously with AICAR and different doses of compound C, a commonly used pharmacological inhibitor of AMPK [15], [16], and found that compound C could block AICAR-induced AMPK activation in a dose dependent manner, and almost extinguish AMPK activation at the highest dose (Fig. 1B). The data indicated AICAR as an activator, and compound C the inhibitor of AMPK, respectively [15], [16]. Moreover, compound C can effectively block AICAR induced AMPK activation.

### Compound C Diminishes LPS-induced AMPK Activation

**Figure 2:**
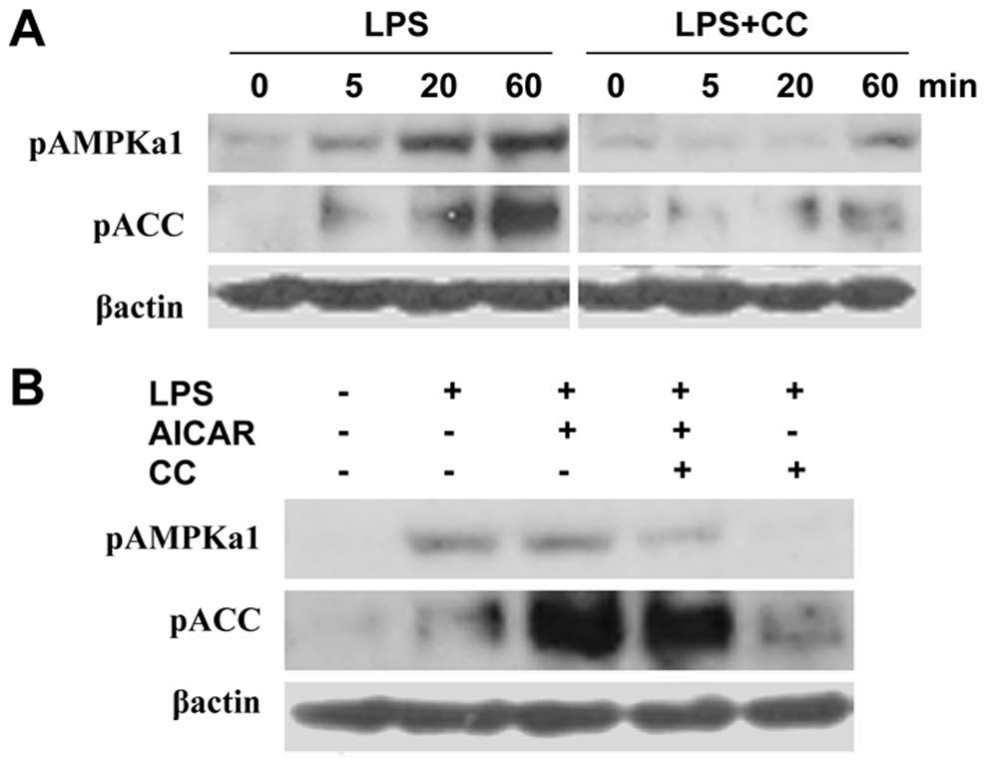
Compound C diminishes LPS induced AMPK activation. **A**. RAW 264.7 cells were pretreated with vehicle or 10 µM compound C (CC) for 15 min, prior to 1 µg/mL LPS challenge. After different time points of LPS challenge, expression of pAMPKα1 and pACC was determined by western blot. **B**. RAW 264.7 cells were treated with vehicle, 1 mM AICAR, 10 µM compound C (CC), or combination of AICAR and compound C for 15 min, followed by 1 µg/mL LPS treatment for 60 min. Expression of pAMPKα1 and pACC was determined by western blot. Data are represented three to four independent experiments.

LPS, the main outer membrane of Gram negative bacteria, contributes to immune response through triggering immediately multiple cell signals [17]. We studied AMPK activation induced by LPS, and noted that LPS initiated phosphorylation of AMPK, as well as its downstream protein, ACC (Fig. 2A). The data above are in accordance with previous studies [18]. Nevertheless, pretreatment of macrophage by compound C diminished LPS-induced phosphorylation of AMPK and ACC (Fig. 2A).

Next, we evaluated the effect of AICAR and compound C on LPS induced AMPK activation. LPS-induced AMPK signaling, especially phosphorylation of ACC, was enhanced by AICAR treatment, whereas AMPK activation induced by the reagents above was inhibited by compound C management (Fig. 2B). The results indicate that compound C as an AMPK inhibitor, can abrogate AMPK activation under a variety of conditions, e.g. AICAR treatment and LPS challenge.

### Compound C Blocks LPS Induced Reactive Oxygen Species (ROS)-NF**κ**B Signaling

**Figure 3:**
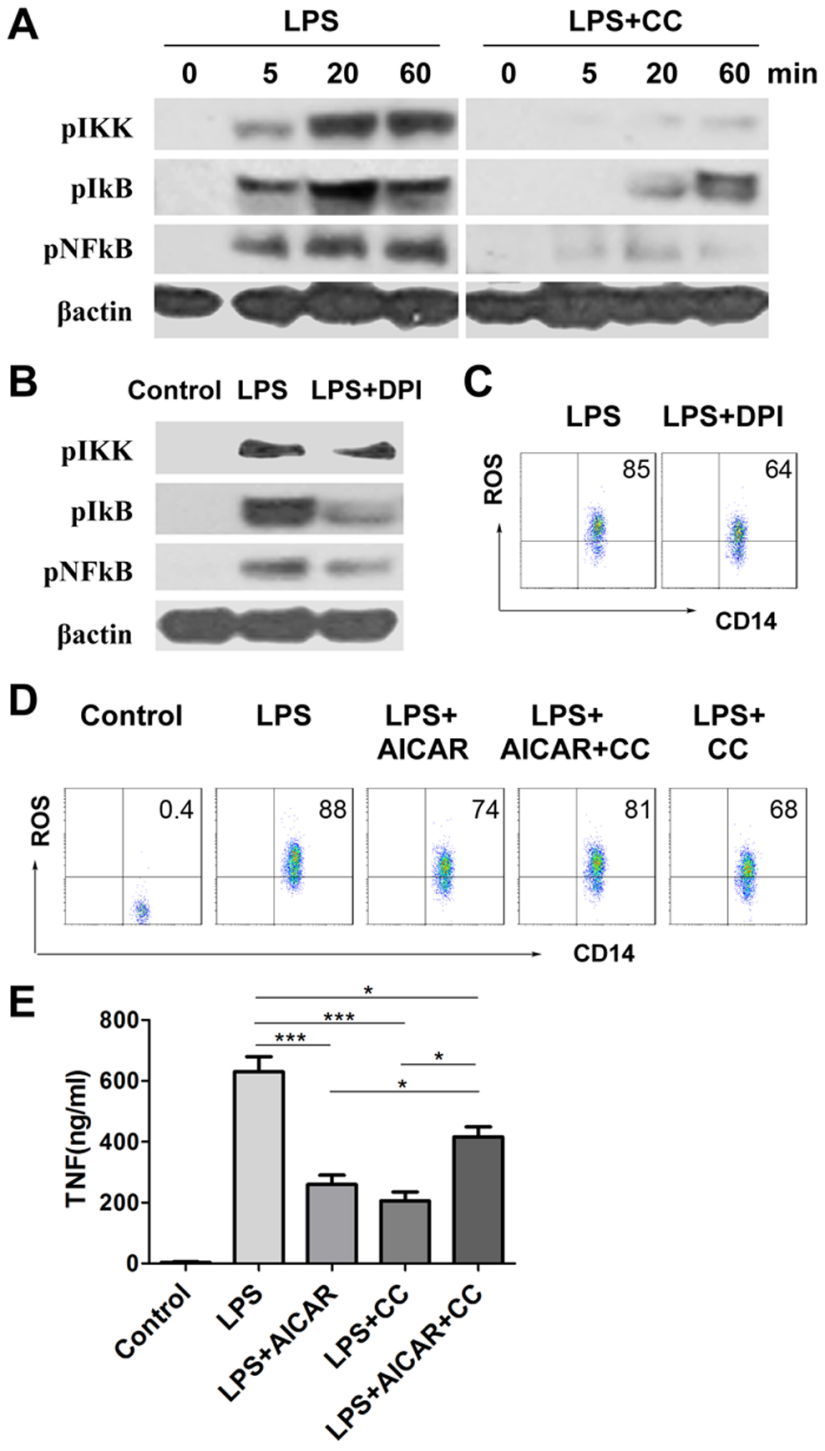
Compound C blocks LPS induced reactive oxygen species (ROS)-NFκB signaling. **A**. RAW 264.7 cells were pretreated with vehicle or 10 µM compound C (CC) for 15 min, prior to 1 µg/mL LPS challenge. Expression of pIKK, pIκB, and pNFκB p65 was determined at different time points after LPS challenge by western blot. **B–C**. RAW 264.7 cells were pretreated with vehicle or 10 µM diphenyleneiodonium (*DPI*) for 15 min, then challenged by vehicle and 1 µg/mL LPS for 60 min (B), or by 1 µg/mL LPS only for 15 min (C). Western blot (B) and flow cytometry (C) was used to determine signaling or ROS generation respectively. **D**. RAW 264.7 cells were pretreated with vehicle, 1 mM AICAR, 10 µM compound C, or combination of AICAR and compound C for 15 min, followed by 1 µg/mL LPS treatment for 15 min. The cells without treatment of LPS and other drugs were set up as Control. ROS generation was analyzed by flow cytometry. **E**. After 15 min pretreatment of vehicle, AICAR or/and compound C as described above, RAW 264.7 cells were stimulated with 100 ng/mL LPS for 18 hours. TNF levels in supernatants were measured by ELISA (n = 4). *, *p*<0.05; ***, *p*<0.005.

We investigated NFκB signal activation inclusive of phosphorylation of IKK, IκB, and NFκB sequentially, and found LPS stimulation on macrophage was characterized by NFκB signal cascade activation (Fig. 3A). It was previously reported that AMPK agonists such as AICAR downregulated LPS induced immune responses [7], [20]. Here we further investigated the effect of AMPK inhibition by compound C on NFκB signaling at different time points after LPS challenge. Pretreatment of compound C dampened phosphorylation of IKK, IκB, and NFκB p65, which were driven by LPS stimulation (Fig. 3A).

Meanwhile, treatment of DPI, the inhibitor of NADPH oxidases (NOX), overrode LPS-induced, phosphorylation of IKK, IκB, and NFκB (Fig. 3B), as well as ROS generation (Fig. 3C), indicating that NOX/ROS mediated LPS-induced NFκB signal activation [19].

To assess ROS induction regulated by AICAR and compound C, we stimulated macrophages with LPS in the presence of AICAR or/and compound C. Both AICAR and compound C inhibited ROS generation (Fig. 3D), NFκB signal activation (data not shown), and TNF production (Fig. 3E). However, combination of AICAR and compound C treatments diminished the inhibitory effect of each single treatment, inclusive of inhibition of ROS generation and TNF production, when compared to LPS alone (Fig. 3D and E).

### Both AICAR and Compound C Attenuate LPS Induced Liver Injury

**Figure 4:**
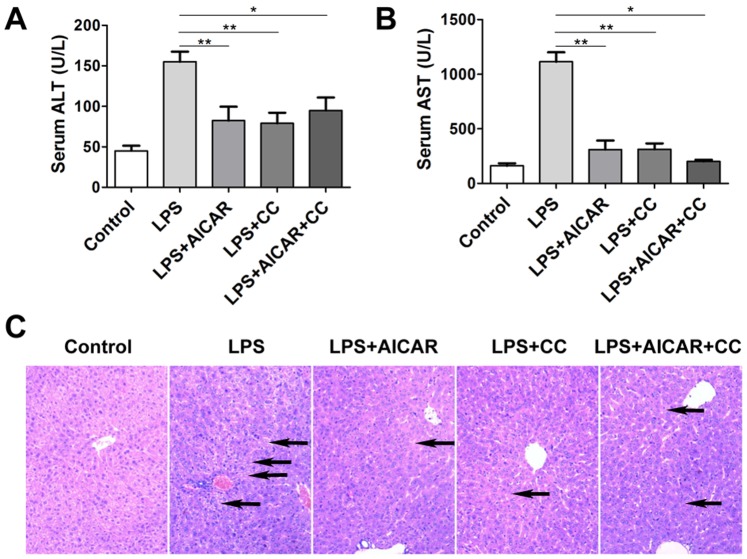
Compound C attenuates LPS induced liver injury. **A–B**. Mice were injected with vehicle as control, LPS (2 mg/kg of body weight), AICAR (500 mg/kg of body weight) plus LPS, compound C (CC, 25 mg/kg of body weight) plus LPS, or AICAR together with CC and LPS as described in Methods. 12 hours after LPS injection, the mice were sacrificed, and blood and live tissue were collected. Serum levels of alanine aminotransferase (ALT) (**A**) and aspartate aminotransferase (AST) (**B**) were measured respectively. **C.** Liver injury was determined by histological examination on HE-stained liver sections, and apoptosis and necrosis of hepatocytes were indicated by arrows (×200 magnification). * *p*<0.05; ** *p*<0.005. Data are represented as mean ± SEM of six to eight independent experiments.

Based on the findings above that both AICAR and compound C blocked LPS induced in vitro macrophage activation, we then hypothesized that AMPK regulators could inhibit LPS-induced immune responses in vivo. Next, we investigated whether these AMPK signaling modulators could protect animals against endotoximia-induced liver injury and death, a typical disease characterized by LPS-induced excessive immune responses [21]. Injection of LPS (i.p.) resulted in liver injury with elevated serum levels of alanine aminotransferase (ALT) and aspartate aminotransferase (AST). However, AICAR (LPS+AICAR) or compound C (LPS+CC) pretreatment decreased the levels of ALT and AST in serum of the mice, in comparison with LPS group (Fig. 4A and B).

We also performed histological assessment of liver injury with hematoxylin and eosin (HE) stained liver sections. As shown in Fig. 4C, endotoximia induced by LPS resulted in hemorrhage or/and congestion in liver tissues, accompanied by dramatic histological changes, including acidophilic degeneration, focal hepatocyte apoptosis, spotty necrosis, and deranged structure of hepatic lobules. Consistent with decreased sera levels of ALT and AST, pretreatments of AICAR or compound C attenuated these histological changes, with minimal occurrence of hepatocyte apoptosis and necrosis (Fig. 4C).

### Compound C Inhibits Immune Responses in Liver

**Figure 5:**
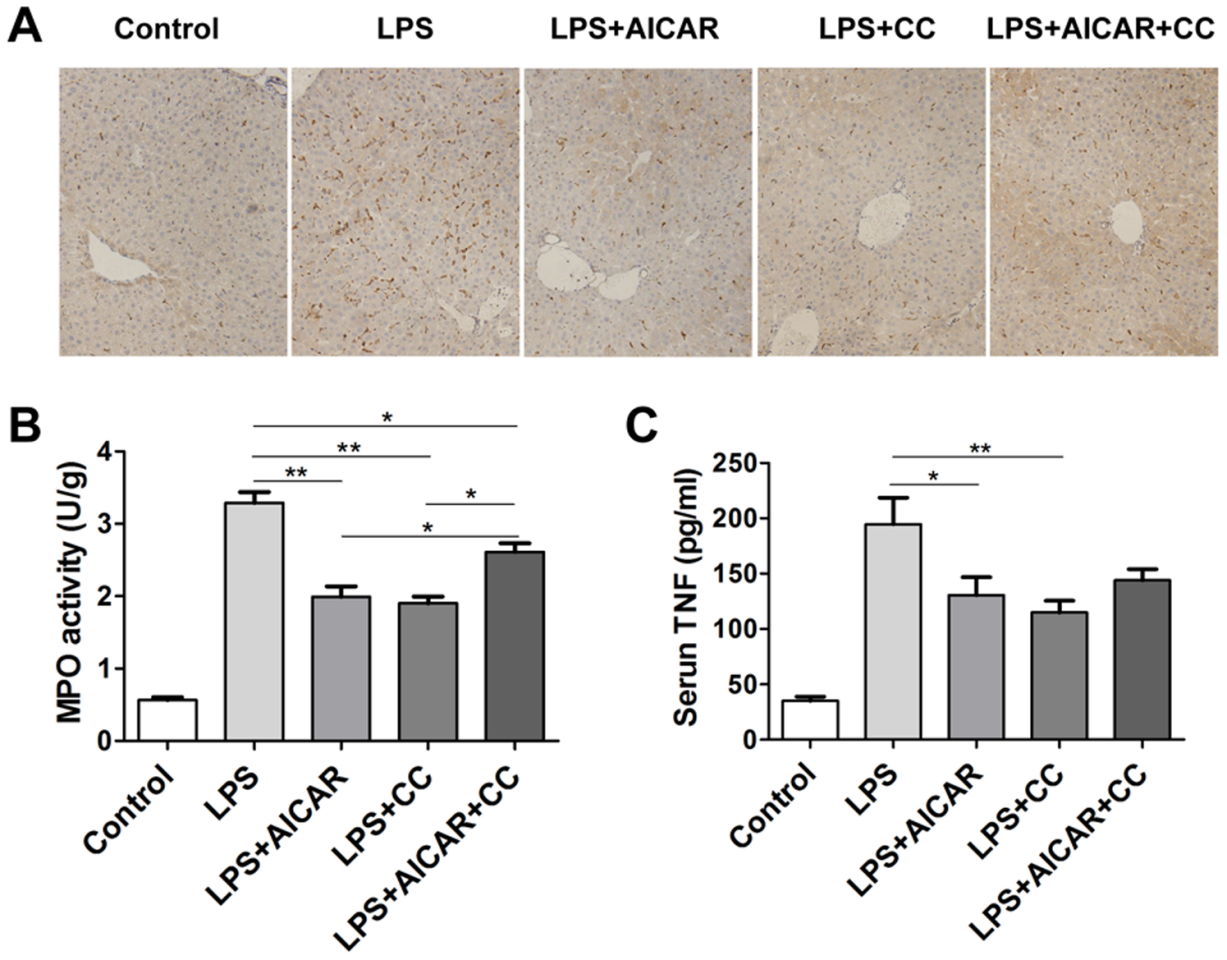
Compound C inhibits immune response in liver. **A–C**. Mice were treated with vehicle as control, LPS (2 mg/kg of body weight), AICAR (500 mg/kg of body weight) plus LPS, compound C (CC, 25 mg/kg of body weight) plus LPS, or AICAR in combination with CC and LPS as described in Methods. 12 hours after LPS injection, the mice were sacrificed, and blood and live tissue were collected. Immunohistochemistry for CD68 (×200 magnification) (**A**), MPO activities (**B**) in liver tissue, and serum TNF levels (**C**) were determined respectively. * *p*<0.05; ** *p*<0.005. Data are represented as mean ± SEM of six to eight independent experiments.

Immune responses including excessive immune cell activation and infiltration into liver tissues contribute to endotoximia induced liver injury [21]. As shown in Fig. 5A, in normal mice, there have a few macrophages (Kupffer cells) distributing in liver local tissues. Intraperitoneal injection of LPS in mice induced rapid inflammatory responses including macrophage and neutrophil infiltration characterized by high levels of CD68 expression (Fig. 5A) and MPO activity (Fig. 5B) in liver tissues, as well as TNF production in serum (Fig. 5C). Treatment of AICAR or compound C inhibited monocyte/macrophage infiltration into liver tissues, with evidence of reduced CD68 expression in liver tissues (Fig. 5A). In accordance with inhibition of TNF production in vitro (Fig. 3E), AICAR or compound C treatment decreased MPO activity in liver tissues (Fig. 5B) and serum TNF production (Fig. 5C), implying that both treatments may also inhibit neutrophil function and infiltration into liver tissues. The data above indicated the negative effect of both single AICAR and compound C treatments on immune responses in liver during endotoximia.

### Compound C Improves Survival of Endotoxemic Mice

**Figure 6:**
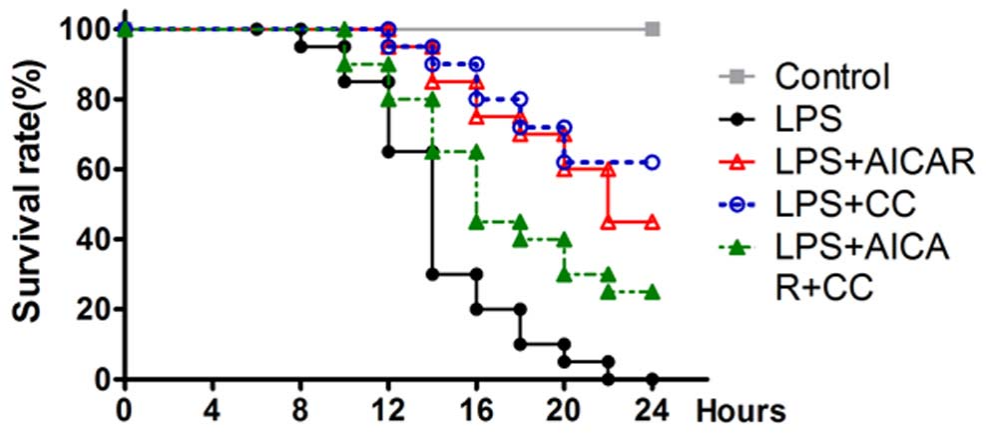
Compound C improves survival of endotoxemic mice. Mice (19–20 animals per group) were administrated with 20 mg/kg of body weight of LPS (filled circles), 500 mg/kg of body weight of AICAR with LPS (open triangles), 25 mg/kg of body weight of compound c (CC) with LPS (open circles), or AICAR plus CC and LPS (filled triangles) as described in Methods. The mice were monitored for lethality every 2 hour for up to 24 hours.

Inhibition of immune responses has been shown to improve survival and protect against endotoximia-induced lethality in experimental animals [22], [23]. We then investigated whether AICAR or compound C could protect against LPS-induced endotoxemic shock and lethality in mice. Treatment of mice with AICAR (LPS+AICAR) or compound C (LPS+CC) before LPS injection significantly reduced lethality in contrast to animals treated with LPS challenge only (*P*<0.01 for LPS+AICAR, and *P*<0.001 for LPS+CC, compared with LPS group), as shown in Fig. 6. Meanwhile, combination treatment of AICAR and compound C (LPS+AICAR+CC) exhibited higher mortality, in contrast to single treatment of AICAR or compound C (Fig. 6).

## Discussion

Endotoximia is a lethal disease with high mortality, which is characterized by excessive activation of immune cells induced by LPS [14]. Generally, LPS, the main potent inflammatory bacterial antigen existing in large quantities in the human digestion tract, is responsible for induction and progression of endotoximia [24]. Under critical clinical conditions such as severe trauma, stroke, and intestinal infection, the components of bacteria i.e. LPS translocate from lumen to intestinal mucosa layer and eventually to portal vein and systemic circulation, resulting in multiple organ dysfunction, especially liver injury [25]. Protection of liver function against endotoximia remains the clinical and experimental challenge for critical care medicine. In the study, we noted that both activator and inhibitor of AMPK signaling attenuate endotoximia-induced immune cell activation, inhibit infiltration of monocytes/macrophages into liver tissues, ameliorate liver injury, and therefore, improve survival of endotoxemic mice. Meanwhile, combination treatment of AICAR and compound C diminishes the inhibitory effects of single treatment of AICAR or compound C.

As an energy sensor, AMPK can be activated by increment in intracellular AMP level, physical or pathogenic stresses, or abnormal energy consumption [9]. Once activated, AMPK acts via its downstream proteins inclusive of ACC, to restore cellular energy levels by stimulating catabolic pathways, e.g. glucose uptake and/or glycolysis and fatty acid oxidation, and curtail ATP-consuming cellular events including synthesis of fatty acids, cholesterol, and protein [26]. Intriguingly, in the study, we have observed that LPS can induce AMPK activation in macrophages, the results may indicate requirement of metabolic demands when immune cells are challenged and activated by LPS and other pathogenic bacterial components. Moreover, pretreatment of compound C abrogated AMPK activation driven by LPS. The data suggest that AMPK signaling is associated with immune cell activation, and might be responsible for energy requirement after cell activation. Meanwhile, inhibition of AMPK by compound C is likely to negate metabolic demands and energy supplement, and diminish LPS induced signaling cascades, cytokine production, and immune cell activation subsequently. However, combination of AICAR and compound C might restore AMPK signaling, and return partly metabolic demands and immune cell activation.

Recently, AMPK has been reported to play an important role in regulating immune responses [14]. We and others have shown that AMPK activators including AICAR and metformin block nuclear translocation of NFκB, and protect animals against inflammatory diseases such as colitis and lung injury [10], [11]. NFκB is one of the pivotal transcription factors in regulation of multiple inflammatory genes including cytokines and chemokines, which can further instigate immune responses [27]. Here we found that, same as AICAR, compound C exhibited negative impact on NFκB signaling, and dampened LPS-induced TNF production in macrophages. The in vivo study also showed that AMPK inhibition by compound C attenuated endotoximia-induced inflammation in liver, and protected mice against endotoximic injury and death. The results suggest AMPK as a regulator in association with NFκB signaling, and both AMPK activator and inhibitor dampen NFκB signaling in immune cells, and abrogate immune responses.

ROS is one of the important intracellular messengers in response to various environmental stimulations [28]. Once generated, ROS regulates plenty of intracellular signal pathways, and modulates cell function and activity [29]. In immune cells, overactivation of ROS signaling can be induced by pathogenic stimuli, and contribute to aberrant cell activation and immune responses of human diseases thereafter [19]. For example, LPS-induce ROS generation and NFκB signaling in macrophages dictate abundant TNF production and the progression of inflammatory diseases, as shown by our present study and other previous findings [19]. We further note that, ROS generation, as well as the downstream cascade signals including IKK, IκB, and NFκB, are diminished by pretreatment of inhibitor and activator of AMPK respectively. The data indicate that AMPK might interact with multiple proteins of ROS-NFκB signaling, and thus dampen activation of these signal proteins.

Compound C has been studied extensively as an inhibitor of AMPK [30]–[32]. Compound C is a selective small molecule AMPK inhibitor in competitive with ATP-binding sites [33]. It was shown that compound C inhibited AMPK activation induced by AICAR or metformin, and reversed the effects of AICAR or metformin, including increment of fatty acid oxidation or suppression of lipogenic genes [34]. This may be the reasons that compound C has been widely used in many studies as the inhibitor of AMPK. Recently, some studies reported that inhibitory effect of compound C was independent of AMPK inhibition, such as suppression of ICAM-1 and VCAM-1 expression in TNF and LPS-treated endothelial cells [35], and inhibition of the hypoxic activation of HIF-1 [36]. However, despite the uncertain specificity of compound C, a great many reports suggest the expected results of AMPK inhibition by compound C in many circumstances [30], [37]. Moreover, the genetic approach has demonstrated that the effect of the AMPK inhibition by compound C was lost in AMPK *α*2 knockout mice during stroke, which confirmed AMPK-specific action of compound C [14], [37]. To date, it has been widely accepted that compound C functions through AMPK inhibition [30]–[32].

Collectively, we report here the pivotal role of AMPK signaling in regulation of immune cell function. Inhibition of AMPK signaling diminishes ROS-NFκB signal cascade, resulting in downregulation of immune responses and attenuation of endotoximia-induced liver injury. Our study may provide AMPK as a possibly therapeutically exploited target for immune disease treatment including endotoxemia and other critical care conditions.
